# Exercise therapy in adults with serious mental illness: a systematic review and meta-analysis

**DOI:** 10.1186/1471-244X-14-117

**Published:** 2014-04-21

**Authors:** Robert Pearsall, Daniel J Smith, Anthony Pelosi, John Geddes

**Affiliations:** 1 Department of Psychiatry, Monklands Hospital, Airdrie, UK; 2 Institute of Health and Wellbeing, University of Glasgow, Glasgow, UK; 3 Regional Eating Disorders Unit, St John’s Hospital, Livingston, UK; 4 Department of Psychiatry, University of Oxford, Oxford, UK

**Keywords:** Exercise, Physical activity, Serious mental illness, Healthy living programme, Physical health

## Abstract

**Background:**

Individuals with serious mental illness are at a higher risk of physical ill health. Mortality rates are at least twice those of the general population with higher levels of cardiovascular disease, metabolic disease, diabetes, and respiratory illness. Although genetics may have a role in the physical health problems of these patients, lifestyle and environmental factors such as levels of smoking, obesity, poor diet, and low levels of physical activity also play a prominent part.

**Methods:**

We conducted a systematic review and meta-analysis of randomised controlled trials comparing the effect of exercise interventions on individuals with serious mental illness.

Searches were made in Ovid MEDLINE, Embase, CINAHL, PsycINFO, Biological Abstracts on Ovid, and The Cochrane Library (January 2009, repeated January 2013) through to February 2013.

**Results:**

Eight RCTs were identified in the systematic search. Six compared exercise versus usual care. One study assessed the effect of a cycling programme versus muscle strengthening and toning exercises. The final study compared the effect of adding specific exercise advice and motivational skills to a simple walking programme. The review found that exercise improved levels of exercise activity (n = 13, standard mean difference [SMD] 1.81, CI 0.44 to 3.18, p = 0.01). No beneficial effect was found on negative (n = 84, SMD = -0.54, CI -1.79 to 0.71, p = 0.40) or positive symptoms of schizophrenia (n = 84, SMD = -1.66, CI -3.78 to 0.45, p = 0.12). No change was found on body mass index compared with usual care (n = 151, SMD = -0.24, CI -0.56 to 0.08, p = 0.14), or body weight (n = 77, SMD = 0.13, CI -0.32 to 0.58, p = 0.57). No beneficial effect was found on anxiety and depressive symptoms (n = 94, SMD = -0.26, CI -0.91 to 0.39, p = 0.43), or quality of life in respect of physical and mental domains.

**Conclusions:**

This systematic review showed that exercise therapies can lead to a modest increase in levels of exercise activity but overall there was no noticeable change for symptoms of mental health, body mass index, and body weight.

## Background

There is now a greater focus on patients’ physical health by mental health services [1]. People with serious mental illness have consistently higher levels of mortality and morbidity than the general population. Mortality rates remain persistently high around twice those of the general population [2]. The life expectancy of people with serious mental illness is shortened by between 11 and 18 years [3]. Despite a steady reduction in mortality rates in the general population no significant change has been observed in people with serious mental illness [4]. The widening differential gap in mortality suggests that people with schizophrenia have not fully benefited from the improvements in health outcomes available to the non-mentally ill population [2].

The underlying causes for the health problems of this population are both complex and multi-factorial [5]. People with serious mental illness such as schizophrenia have higher levels of cardiovascular disease [6,7], metabolic disease [8], diabetes [9,10], and respiratory illness [11,12]. Although genetics may have a role in the physical health problems of these patients, lifestyle and environmental factors such as smoking, obesity, poor diet, and low levels of physical activity play a prominent part [13]. Some of the treatments given to people with serious mental illness contribute to the health problems in this population. For example, neuroleptic medication can lead to significant metabolic problems such as weight gain, lipid abnormalities, and changes in glucose regulation [14]. Evidence is becoming clearer that long term exposure to these medications may contribute to the higher mortality levels in this population [5].

There is now a greater focus on attempting to improve the physical health of these patients. However they have less access to medical care, poorer quality of care, and preventative health checks are less commonly completed in both primary and secondary care compared with the general population [15-17]. The nature of their mental illness may also affect their motivation as many individuals may be not ready to change their lifestyle [18,19]. Individuals with serious mental illness have higher levels of smoking, weight problems, poor dietary intake, and low levels of physical activity. Rates of smoking of up to 70% have been found in patients with schizophrenia [7,20,21]. The prevalence of smoking in the general population is approximately 20% [22]. Individuals with serious mental illness have a poorer diet than the non-mentally ill population [20,23]. Levels of obesity range from 40-60%, up to four times that of the non-mentally ill population [24-26]. These individuals are less active with lower levels of physical activity compared with the general population [27-29].

Regular physical activity has been found to be beneficial for both physical and mental health in the general population. There is a direct relationship between physical activity and a reduction in cardiovascular disease, cerebrovascular disease, stroke, and hypertension [30,31]. Physical activity also leads to a reduction in the risk of metabolic health problems such as diabetes and metabolic syndrome [30]. A routine level of at least 150 minutes of physical activity per week is required to achieve a consistent reduction in risk [31]. In the general population about 60% of men and 70% of women self-report less than the recommended levels and objective measures of activity suggest that far more of these individuals are failing to meet the recommendations [32]. Physical activity has beneficial effects on mental health. It has been shown to be effective in the treatment of depression [33] and anxiety disorders [34]. It has positive effects on psychological well-being [35], quality of life [36] and in the reduction of stress [37].

The aim of this review was to determine the effectiveness of exercise programmes for people with serious mental illness. Two main objectives of this review were to firstly determine the effect of these programmes on levels of exercise activity, and secondly the effect of exercise on mental health and well-being.

## Methods

### Eligibility criteria

Studies met the following criteria for inclusion in the review:

1. Adults with schizophrenia or other types of schizophrenia-like psychosis, schizoaffective disorders, and bipolar affective disorder irrespective of the diagnostic criteria used, age, ethnicity and sex.

2. All patients, adults, clients, in the community or in hospital.

3. All relevant randomised controlled trials.

4. Interventions where a primary or secondary aim was to promote exercise or physical activity.

### Search Methods, and study selection

We searched the following electronic databases: Ovid MEDLINE, Embase, CINAHL, PsycINFO, Biological Abstracts on Ovid, and The Cochrane Library (January 2009, repeated May 2013). The systematic search included hand searching of journals, books, cross-referencing and bulletins (e.g. brief reports/brief statement of facts). The search filter, the Cochrane Highly Sensitive Search Strategy, was used to assist in the identification of randomised trials in MEDLINE [38].

The abstracts of studies were examined by RP. Full text of the studies that potentially met the eligibility criteria was obtained. Discrepancies were discussed with co-investigators. We checked articles that met the inclusion criteria for duplication of the same data.

### Data extraction and analysis

Data was extracted by one author (RP) and checked for accuracy by the second (DS). Data was extracted onto prepared forms to include: participants and setting, location, description of the intervention, type of exercise, study size, methodological issues, risk of bias, results, and general comments. All analyses were conducted using Revman Manager version 5.1. We performed a PRISMA evaluation of our meta-analysis using a standard checklist of 27 items that ensure the quality of a systematic review or meta-analysis [39]. A summary measure of treatment effect was used as different outcome measures were found. The standardized mean difference (SMD) with 95% confidence intervals was calculated as the difference in means between groups divided by the pooled standard deviation. If no standard deviations were found they were calculated from standard errors, confidence intervals, or t values [40]. Authors were contacted for missing data if analyses could not be completed. Statistical heterogeneity was assessed by the I^2^ test. The degree of heterogeneity was categorised as the following [36]: 0% to 40% low level of heterogeneity; 30% to 60% moderate heterogeneity; 50% to 90% substantial heterogeneity; 75% to 100%: considerable heterogeneity. Standard mean differences were based on the random-effects model as this would take into account any differences between studies even if there was no statistically significant heterogeneity [40].

### Quality assessment

There is no agreed standardised method to assess the quality of studies in systematic reviews. We adapted the Cochrane Collaboration’s tool for assessing the risk of bias [40]. The following recommended domains were considered: sequence generation, allocation concealment, blinding, incomplete outcome data, selective outcome reporting, and other sources of bias. Each item was rated according to the level of bias and categorised into either low, high, or unclear. The category unclear indicated unclear or unknown risk of bias [40].

## Results

**Figure 1:**
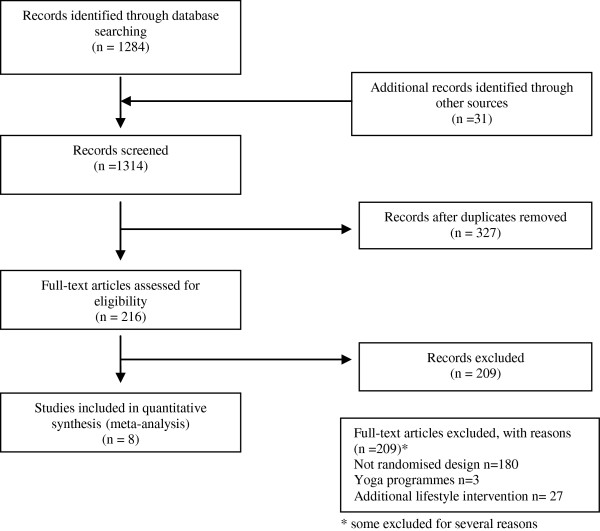
Flowchart of the results of the systematic search.

The electronic search identified 1284 potentially eligible reports. Nine hundred and thirty two were excluded on the basis of the title or abstract alone. We retrieved the full text of 216 articles and excluded a further 209 studies. The review excluded a large number of studies as they included additional components such as dietary or weight programmes (Figure 1).

All included studies had been published between 2005 and 2013. The studies varied in their setting, size, age, study intervention type, and use of outcome measures.

### Study characteristics

Characteristics of the sample, interventions, outcomes assessment and results are shown in Tables 1, 2 and 3.

**Table 1:** Systematic review – exercise interventions (Description of programmes)

**Study**	**Study outline**	**Description of intervention**
Beebe et al. [41]	Study to assess the effectiveness of an exercise programme on the physical and mental health of individuals with schizophrenia.	Intervention to determine whether an exercise programme can improve the physical and mental health of individuals with schizophrenia.
		12 participants, random allocation to two groups, intervention and control group (randomisation schedule by statistician). 16 week intervention.
		Intervention group – treadmill exercise programme, meeting 3 times/week for 16 weeks. Programme consisted 10 minutes warm-up session, treadmill walking, then 10 minutes of cool-down exercises. Treadmill walking session gradually increased from 5 minutes to 30 minutes per episode for rest of study.
		Control Group – no exercise programme offered to this group, until end of study.
		Attendance – 43% to 91% of total sessions offered, 75% attended half of sessions, 50% attended 2/3.
		Assessments – demographics, weight/ height/ BMI, 6-Minute Walking Distance (6 MWD), Percentage Body Fat, Positive and Negative Syndrome Scale Scores (PANSS).
Skrinar et al. [45]	Study to assess the impact of an exercise programme in individuals with serious mental illness.	Intervention of patients with mood and psychotic illness. To determine the effect of a 12 week exercise programme on physical and mental health measures.
		12 week programme. 2 groups - intervention group and control group. Patients randomly assigned.
		Intervention group – 4 exercise per week for 12 weeks + one health seminar per week. Aerobic and cardiovascular training and cool-down each session, increasing intensity of exercise. Health seminar topics – healthy eating, weight management, stress relief, spirituality and wellness.
		Control group – normal care, although offered exercise programme end of study + asked to keep record of amount of exercise they will do during control phase.
		Assessments – demographics, weight, height, pulse, blood pressure, blood tests, Symptom Checklist- 90- R, Lehman Quality of Life Questionnaire, Boston University Making Decisions Questionnaire, MOS 36- Item Short-Form Health Survey (SF- 36). Pre and post-intervention assessment.
Acil et al. [47]	Study to determine the of 10 week exercise programme v. control, in patients with schizophrenia.	10 week programme using intervention and control group. Mixed group of inpatients and outpatients. Randomised two groups.
		Intervention group – 10 week, 3 days per week, and 40 min. per intervention session.
		Aerobic exercise programme for first 2 weeks with 25 min. per day. Starts with 10 min. work up then 25 min. aerobic exercise. Finally 5 min. cooling down.
		Control group – no details of control group.
		Baseline assessment – following assessments:- demographic data, heart rate, Scale for Assessment for Negative Symptoms (SANS), Scale for the Assessment of Positive Symptoms (SAPS), Brief Symptom Inventory (BSI), World Health Organization Quality of Life Scale-Turkish Version (WHOQOL-BREF-TR).
Marzolini et al. [46]	Study to assess the effectiveness of an exercise programme for individuals with schizophrenia.	Intervention to determine the efficacy of a group-based exercise programme. Multidisciplinary approach using a resistance and aerobic exercise programme.
		Intervention over 12 weeks. 2 groups, exercise group and usual care (control). Randomised allocation.
		Intervention – exercise twice per week for 12 weeks at local recreation centre + once per week additional aerobic exercise session individually or during home-visit from mental health clinician. Advised to exercise to same pace and duration at home.
		Control Group – “usual care” continued. No other intervention except measurement at baseline and 12 weeks.
		Assessments – demographic history, weight/BMI, waist/hip circumference, resting blood pressure, functional exercise capacity (6-Minute Walking Distance), muscular strength (one repetition maximum test), anthropometric measurements, adherence to exercise tests, Mental Health Inventory (MHI), self designed feedback questionnaire.
Beebe et al. [43]	Study to determine the effect of effect of exercise advice and techniques, motivational interviewing skills, and a walking programme compared with the control comprising a walking group only in people with schizophrenia spectrum disorders.	Randomised controlled trial of an intervention to effect of exercise advice and techniques, motivational interviewing skills, and a walking programme compared with the control, comprising a walking group only - in people with schizophrenia spectrum disorders. 16 week walking programme with intervention and control group. WALC-P versus TAC
		Intervention – WALC-S programme – **W**alk (discuss walking/information/advice), **A**ddress Sensations (discussion about discomforts warming up or cooling down, suggestions to minimise problems), **L**earn about Exercise (information on exercise benefits and barriers), **C**ue Exercise (calendars or times to start walking, with reminder calls etc.).
		Control group – TAC group (time and attention control) consisted of 4 weekly, 1 hour (8–9 subjects per group) – focussing on health behaviour, smoking, relaxation, medication adherence.
		All patients attended walking group for 16 weeks. Assessments – demographic data, walking group attendance, walking group persistence and compliance.
Methapatara et al. [44]	Study to examine the effects of a walking programme in individuals with schizophrenia.	Study to compare the effects of a walking programme combined with motivational interviewing in patients with schizophrenia who are overweight or obese.
		Randomised open label, parallel controlled trial of 12 week duration. Patients with schizophrenia with a BMI of 23 kg/m2. Intervention compared with the control in ratio 1:1. Programme started pre-discharge in hospital for 1 week. Advised to increase walking post-discharge.
		Intervention Group – five 1 hour sessions. Individual motivational interviews given at first session focussing on adequate daily walking. Second session involved group education on nutrition, exercise, warming up, cooling down, and start of use of pedometers. Minimum of 3000 steps per day recommended level of walking.
		Fourth session group walking. Individuals encouraged to increase walking at other times. Fifth session – feedback on programme/progress.
		Control Group – received usual care only. No pedometer given.
		Assessments demographics,, body weight, waist circumference, CGI-Severity, MMSE-Thai.
Scheewe et al. [42]	Study to assess the effectiveness of exercise therapy on cardiorespiratory fitness (CRF) in individuals with schizophrenia.	Intervention to study the effect of an exercise intervention on CRF in patients with schizophrenia compared with matched controls. Trial part of the TOPFIT project. Twenty four week programme. Sixty-three patients with schizophrenia and 55 controls randomised. Randomisation computer generated, stratified for gender, recruitment site and BMI. Four centres, community recruitment.
		Intervention – 1:1 allocation of exercise or occupational therapy. Exercise consists of cycling, rowing, hiking and muscle exercises. Two times a week for 1 hour. Occupational therapy consisted of computer operations, drawing, sculpting and painting.
		Control group - exercise or life as usual. Assessments – Scheewe et al., 2012: CRF assessed using a Cardiopulmonary exercise test (CPET) – stepwise incremental protocol to exhaustion on an upright cycle ergometer. Terminated at voluntary exhaustion. Physiological measurements – blood pressure, heart rate, oxygen update, peak minute ventilation. Scheewe et al., 2013: PANSS, MADRS, Camberwell Assessment of need (CAN), body mass index, waist circumference, blood pressure, blood parameters. Scheewe et al., 2013: Global brain volumes, hippocampal volume, and cortical thickness.
Pelham et al. [48]	Study to assess the impact of an exercise programme in individuals with serious mental illness.	Intervention to assess the effect of an exercise programme using cycling as the method of exercise activity compared to a control. 12 week programme. Eight patients with schizophrenia and 2 with bipolar affective disorders. Total 10 patients in programme. Randomisation into two equal groups. Based in psychiatric rehabilitation services.
		Intervention – exercise activity on a cycle ergometer. Four sessions per week for 30 minutes at 65% to 75% of heart rate reserve.
		Control – muscle tone and strengthening exercises, 4 times per week for 30 minutes each session. Short intermittent bouts used within this to keep heart rate below 110 beats per minute.
		Assessments – VO_2_ max tests, Becks Depression Inventory, and weight measurements.

**Table 2:** Systematic review – exercise interventions (Methodological issues/Risk of bias)

**Study**	**Country**	**Participants & setting**	**Design**	**Recruitment**	**Baseline sample**	**Number completing trial**	**Methodological issues/Risk of bias**
Beebe et al. [41]	USA	Patients attending outpatient clinic at a Veterans Hospital	RCT	Volunteers	12	10	Participants – eligibility criteria specified - yes
							- standardised diagnostic criteria - Yes
							- non-random recruitment
							- similar baseline groups
							Intervention – details of therapists training not indicated
							- no treatment manual
							- compliance of medication non- checked
							Measurement of Outcome
							- outcome assessors blinded to treatment allocation
							- adequate follow- up period
							Risk of Bias - adequate sequence generation- unclear
							allocation concealment- unclear
							blinding of participants, personnel and outcome assessors - yes
							incomplete data outcome - yes
							free of selective outcome reporting- yes
							free of other sources of bias- yes
							Small sample, majority male, control group awareness of exercise programme.
							Attendance at sessions variable.
Skrinar et al. [45]	USA	Patients from inpatient, partial hospitalisation, outpatient and community centres in area of McLean Hospital.	RCT	Selection/ invitation	30	20	Participants – eligibility criteria specified - yes
							- standardised diagnostic criteria -yes
							- non-random recruitment
							- similar baseline groups –
							Intervention – details of therapists training not indicated
							- no treatment manual
							Measurement of Outcome
							- outcome assessors not blinded to treatment allocation
							- adequate follow- up period
							Risk of Bias - adequate sequence generation - unclear
							allocation concealment - unclear
							blinding of participants, personnel and outcome assessors - no
							incomplete data outcome - no
							free of selective outcome reporting - yes
							free of other sources of bias - yes
							Small sample, variable adherence to programme and attendance, inclusion criteria include mood disorders as well.
							Control group may be affected by exercise “influence” of study.
Acil et al. [47]	Turkey	Inpatient and outpatient. Diagnosis of schizophrenia	RCT	Recruitment method not described, 30 outpatients and inpatients.	30	30	Participants – eligibility criteria poorly specified
							- standardised diagnostic criteria- yes
							- non-random recruitment
							- similar baseline group
							Intervention – details of therapists training not indicated
							- no treatment manual
							Measurement of Outcome
							- outcome assessors not blinded
							- adequate follow- up period
							Risk of Bias - adequate sequence generation - no
							allocation concealment - no
							blinding of participants, personnel and outcome assessors - no
							incomplete data outcome - unclear
							free of selective outcome reporting - unclear
							free of other sources of bias - yes
							Non-standardized exercise intervention. No measurement of existing exercise level or patient participation in the programme.
							Number completed trial not given.
Marzolini et al. [46]	Canada	Identified from Community Mental Health Programme. Majority lived supported accommodation.	RCT	Volunteers	13	13	Participants – eligibility criteria specified - yes
							- standardised diagnostic criteria -yes
							- non-random recruitment
							- similar baseline groups –
							Intervention – details of therapists training not indicated
							- no treatment manual
							Measurement of Outcome
							- outcome assessors not blinded to treatment allocation
							- adequate follow- up period
							Risk of Bias - adequate sequence generation - yes
							allocation concealment - yes
							blinding of participants, personnel and outcome assessors - unclear
							incomplete data outcome - no
							free of selective outcome reporting - yes
							free of other sources of bias - yes
							Small sample, participants supported accommodation. Inclusion criteria – patients had to have one or more cardiovascular risk factors.
Beebe et al. [43]	USA	Outpatients	RCT	Volunteers	97	79	Participants – eligibility criteria specified
							- standardised diagnostic criteria
							- non-random recruitment
							- similar baseline groups
							Intervention – details of therapists training indicated
							- treatment manual - yes
							- compliance of medication non- not checked
							Measurement of Outcome
							- outcome assessors not blinded to treatment allocation
							- adequate follow- up period
							Risk of Bias - adequate sequence generation - yes
							allocation concealment - unclear
							blinding of participants, personnel and outcome assessors - unclear
							incomplete data outcome - unclear
							free of selective outcome reporting - yes
							free of other sources of bias - yes
							Well-designed programme. Adequate sample size.
							No assessment of mental health changes - improvement in mental health may have contributed to increase in exercise by itself.
Methapatara et al. [44]	Thailand	Inpatient & Outpatients	RCT	Volunteers	64	64	Participants – eligibility criteria specified
							- standardised diagnostic criteria – not indicated
							- volunteers
							- dissimilar baseline groups –
							younger control population
							Intervention – details of therapists training not indicated
							- treatment manual - no
							- compliance of medication not checked
							Measurement of Outcome
							- outcome assessors blinded to treatment allocation
							- adequate follow- up period
							Risk of Bias - adequate sequence generation - yes
							allocation concealment - yes
							blinding of participants, personnel and outcome assessors - no
							incomplete data outcome - yes
							free of selective outcome reporting - yes
							free of other sources of bias - yes
							Small sample size, no record of daily steps recorded or measurement of change in exercise levels. Compliance with programme unknown (as no recording of pedometer). No assessment of nutritional intake which may have affected outcome. Effect on mental health unknown.
Scheewe et al. [42]	USA	Community	RCT	Volunteers	118	92	Participants – eligibility criteria specified - yes
							- standardised diagnostic criteria – not indicated
							- non-random recruitment
							- dissimilar baseline groups
							Intervention – details of therapists training indicated
							- treatment manual
							Measurement of Outcome
							- outcome assessors not blinded to treatment allocation
							- adequate follow-up period
							Risk of Bias - adequate sequence generation - yes
							allocation concealment - yes
							blinding of participants, personnel and outcome assessors - no
							incomplete data outcome - unclear
							free of selective outcome reporting - yes
							free of other sources of bias - yes
							Well-designed programme. Large sample size. Computer generated randomisation. Study part of larger research trials. No results from mental health assessment. Baseline group differences. Mean weight greater in patients than controls. Motivated group of patients with higher level of fitness at baseline. No follow up of participants to show whether improvement in exercise maintained.
Pelham et al. [48]	USA	Community Rehabilitat-ion	RCT	Volunteers	10	10	Participants – eligibility criteria specified - yes
							- standardised diagnostic criteria – not indicated
							- non-random recruitment
							- baseline groups unclear
							Intervention – details of therapists training indicated - no
							- treatment manual- no
							Measurement of Outcome
							- outcome assessors not blinded to treatment allocation
							- adequate follow-up period
							Risk of Bias - adequate sequence generation - unclear
							allocation concealment - unclear
							blinding of participants, personnel and outcome assessors - no
							incomplete data outcome - unclear
							free of selective outcome reporting - unclear
							free of other sources of bias – unclear
							Small study. Control also using exercise activity. Data not available to include in meta-analysis. Early study 21 years ago. Beneficial effects on exercise fitness and depression scores.

**Table 3:** Systematic review – exercise interventions (Results/Comments)

**Study**	**Results**	**Comments/Analysis**
Beebe et al. [41]	6-Minute Walking Distance (MWD) – improvement in distance clinically (152.5 mins) compared with the control group (56.7 mins), but not statistically significant. BMI and body fat reduced in intervention group compared with the control group but not statistically significant. Control Group – increased 6- MWD during intervention (5%).	Valuable study, limited by small sample, population type. Control group showed some increase in physical activity (? overlap effect of intervention).
Skrinar et al. [45]	Weight change intervention: control -2.2 v. -1.2 kg (non-significant). Exercise intensity increased in intervention compared with the control + 8 Watts v. -5 Watts (non-significant). Significant improvement in results in general health (p < 0.05) and empowerment (p < 0.01).	Valuable study although some limitations in methodology.
Acil et al. [47]	Exercise programme resulted in decrease in psychiatric symptoms and increase in quality of life. Reduced SANS, SAPS and BSI. Increase in WHOQOL-BREF-TR. No exercise measures change of heart rate. Demographic data not provided.	Valuable intervention study, however limited by small sample size, and lack of standardization of intervention. No details of drop-out rate or measurement of exercise levels in subjects.
Marzolini et al. [46]	Mean age 43 years. Exercise group showed a 27.7 metre (SD ± 22.3 m) increase in 6MWD while control group showed decrease of 28.3 metres (SD ± 26.6 m) (between group difference, p = 0.1). There was significant increase in strength exercise and Mental Health Inventory. Attendance averaged 72% (SD ± 4.4%) with no dropouts.	Valuable study but limited by small sample size. Good adherence to programme.
Beebe et al. [43]	Percentage attendance in WALC-S group 35.2 versus 27.3% after 16 week programme. 33.7 versus 22.9 in TAC group. Greater persistence in weeks in WALC-S versus TAC, and higher minutes walked (76.67 versus 116.89) in WALC-S group, and (61.88 versus 788.83) in TAC group.	Well-designed study indicating the benefit of exercise advice with a motivational programme in addition to exercise intervention.
Methapatara et al. [44]	End of programme, mean body weight decreased significantly compared with the control group by 2.21 kg (p = 0.03).	Valuable study showing the benefit of an exercise programme in overweight or obese patients with schizophrenia.
Scheewe et al. [42]	Patients had higher resting HR, lower pear HR, peak systolic BP, relative VO_2 peak_, W_peak_, RER, minute ventilation, and HR recovery than controls. In conclusion patients had lower CRF levels compared with controls. Exercise therapy increased VO_2 peak_, and W_peak_ in patients and controls. VO_2 peak_, and W_peak_ decreased in non-exercising patients.	Well-designed study. Results show an increase in CRF in individuals with schizophrenia. However individuals may be more motivated and have greater baseline fitness than many individuals with this illness.
	Trend-level effect on depressive symptoms (p = 0.07). No effect on symptoms of schizophrenia.	
	Significantly smaller baseline cerebral (gray) matter, and larger third ventricle volume, thinner cortex. NO change global brain, hippocampal volume, or cortical thickness.	
Pelham et al. [48]	Aerobic exercise group showed significant increases in fitness and a reduction in depression scores. Non-aerobic groups did not improve in fitness level or level of depression.	Small study of 10 patients. Conducted 21 years ago. Findings showed the possible positive effects of exercise on levels of fitness and mental health in people with serious mental illness.

### Setting and participant characteristics

Eight studies met the inclusion criteria. Of these 5 were based solely in either a community or outpatient setting. The remainder comprised a combination of inpatient hospital together with community or outpatient programmes. Diversity of setting may have had an impact on the delivery and generalisability of the exercise interventions. Exercise change may be more easily achievable within a more closely supervised setting such as an inpatient ward. The sample size of studies in this review varied considerably. The largest study had 118 patients in their programme the smallest 10 individuals. The mean age of individuals ranged from 27 years to 52 years. The majority were in the age group 30–40 years. One study [41] used an older age population with a mean age of 46.9 years and one a younger group with a mean of 29.2 years [42]. The variation in age group may have affected the implementation of the programme. A younger population may have been more able to improve their level of exercise. The ethnicity of participants was described in only two studies. Beebe et al. [43] found that 54% of participants were Caucasian, 44% African-American, while Beebe et al. [41] in a small study of 10 participants, 80% were Caucasian and 20% African-American.

### Exercise interventions

Each study used a different type of aerobic exercise incorporating cardiovascular exercise and resistance training. Three studies used walking as their exercise activity [41,43,44], while four studies used a combination of general aerobic and cardiovascular exercise [42,45-47]. Cycling was the method of exercise activity used in the remaining study [48]. In addition to the exercise activity general information was given to all participants about exercise. Only two studies used more specific advice and guidance [43,45]. Skrinar et al. [45] offered seminars on a range of topics such as adequate individual levels of exercise, healthy eating, stress relief, spirituality and wellness. In the remaining study Beebe et al. (2011) compared the effect of adding specific exercise advice and motivational skills to a simple walking programme [43].

Only one study [42] used a standardised programme of exercise comprising of cardiovascular and muscle strength exercises [49]. Moderate levels of exercise intensity were described in 7 out of the 8 programmes. Only Methapatara et al. [44] described the specific amount of exercise activity used in their programme. The actual amount or dose of exercise activity was not measured in the remaining studies.

Each programme varied in their frequency and duration. Some were twice a week [46] while Skrinar et al. [45] described a programme 4 times per week. The duration of the programmes lasted between 10 weeks [47] to 24 weeks [42].

### Outcomes

A variety of different outcome measures were used (Table 4). This made it especially difficult to compare the results of individual interventions. Two studies used a validated measure of exercise, namely the 6-Minute Walking Distance [41,46]. Others used measures such as body mass index [46], the number of exercise sessions attended [45], or the number of minutes walked [43].

**Table 4:** Comparison: exercise versus standard care

**Outcome or subgroup title**	**No. of studies (available data)**	**No. of participants**	**Statistical method**	**Effect Size (SWD)**
Exercise activity	1	13	SMD (IV, Random, 95% CI)	1.81 [0.44 to 3.18]
BMI	4	151	SMD (IV, Random, 95% CI)	-0.24 [-0.56 to 0.08]
Weight	2	77	SMD (IV, Random, 95% CI)	0.13 [-0.32 to 0.58]
Negative	2	84	SMD (IV, Random, 95% CI)	-0.54 [-1.79 to 0.71]
Symptoms				
Positive	2	84	SMD (IV, Random, 95% CI)	-1.66 [-3.78 to 0.45]
Symptoms				
Anxiety/Depression	3	94	SMD (IV, Random, 95% CI)	-0.26 [-0.91 to 0.39]
Q of L (Physical)	2	30	SMD (IV, Random, 95% CI)	0.45 [-0.27 to 1.18]
Q of L (Mental)	2	30	SMD (IV, Random, 95% CI)	0.65 [-0.09 to 1.39]

**Figure 2:**
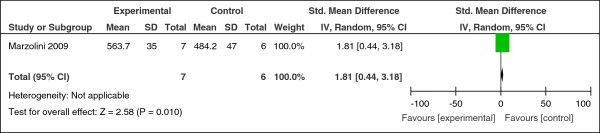
Effect of exercise versus usual care: 6-minute walking distance.

**Figure 3:**
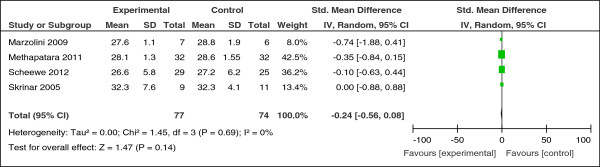
Effect of exercise versus usual care: body mass index.

**Figure 4:**
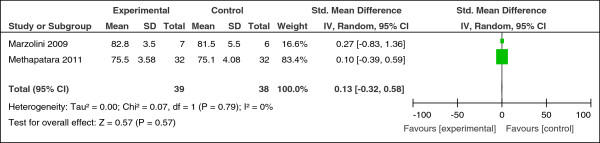
Effect of exercise versus usual care: weight.

Six studies compared the effect of exercise with usual care. A significant increase in the distance walked in 6 minutes was found in one RCT (n = 13, SMD = 1.81, CI 0.44 to 3.18, z = 2.58, p = 0.01) (Figure 2) [46]. A small non-significant reduction was found on body mass index (n = 151, SMD = -0.24, CI -0.56 to 0.08, p = 0.14; heterogeneity, Chi^2^ = 1.45, I^2^ = 0%, p = 0.69) (Figure 3). No effect was found comparing the effect of exercise on body weight (n = 77, SMD = 0.13, CI -0.32 to 0.58, p = 0.57; heterogeneity, Chi^2^ = 0.07, I^2^ = 0%, p = 0.79) (Figure 4).

**Figure 5:**
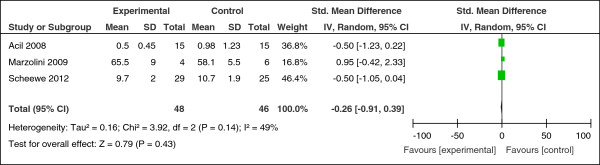
Effect of exercise versus usual care: anxiety & depression.

There was no overall beneficial effect on negative symptoms (n = 84, SMD = -0.54, CI -1.79 to 0.71, p = 0.40) or positive symptoms of schizophrenia (n = 44, SMD = -1.66, CI -3.78 to 0.45, p = 0.12). Exercise did not lead to an improvement in anxiety and depressive symptoms (n = 94, SMD = -0.26, CI -0.91 to 0.39, p = 0.43; heterogeneity, Chi^2^ = 3.92, I^2^ = 49%, p = 0.14) (Figure 5).

Data from single RCTs was available for the following outcome measures. Overall, there was no clear evidence that exercise interventions led to significant improvements in quality of life. A small non-significant increase in points of physical and mental domains were found (physical domain: n = 30, SMD = 0.45, CI -0.27 to 1.18, z = 1.22, p = 0.22: mental domain: n = 30, SMD = 0.65, CI -0.09 to 1.39, z = 1.73, p = 0.08).

The final study compared the effect of adding specific exercise advice and motivational skills to a simple walking programme [43]. No significant change was found in the attendance at walking groups, persistence, or minutes walked compared with the control.

### Methodological design and quality

**Figure 6:**
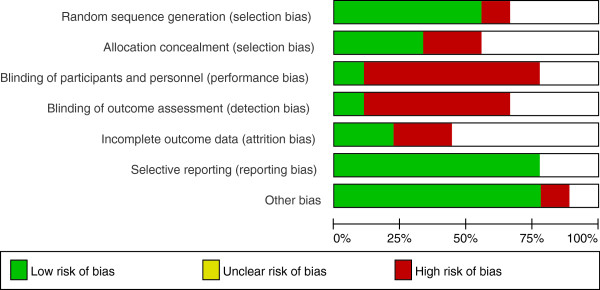
Risk of bias graph: review authors’ judgements about each risk of bias item presented as percentages across all included studies.

The review found a lack of standardisation in terms of the exercise intervention, setting and outcomes measures (Tables 1, 2 and 3). Six studies described an adequate method of randomisation. Four out of the 8 studies used satisfactory methods to conceal the allocation of treatment. Beebe et al. [41] was the only study to incorporate outcome assessors blinded to the treatment group. There was considerable variation in sample size. No study described a sample size calculation. We used the Cochrane Collaboration’s tool for assessing the risk of bias [40] (Figure 6).

One study only adequately addressed the analysis of incomplete data [44]. Data was analysed on an intention-to-treat basis with last observation carried forward on the following measures, bodyweight, body mass index, and waist circumference.

Levels of attrition varied in the included studies between 0% and 33% with a mean of 18.9%. No dropout was observed in two studies [44,46]. Methapatara et al. [44] had no attrition in a larger study of 64 individuals, perhaps suggesting that participants in this study were more motivated to change.

## Discussion

Exercise programmes found in this review had a modest beneficial effect on levels of exercise activity. Exercise did not lead to an improvement in body mass index, negative or positive symptoms of schizophrenia, or the individual’s quality of life. No effect was found on body weight or symptoms of anxiety or depression. No beneficial effect was found by the addition of specific exercise advice with motivational techniques to a simple exercise programme. The review found that many studies varied in the type of exercise programme used, setting, age group, sample size, and outcome measures. Comparison of these outcomes proved difficult. Diversity of setting may have had an impact on the delivery and generalisability of the exercise interventions. Exercise change may be more easily achievable within a more closely supervised setting such as an inpatient ward.

The exercise programmes used a variety of outcome measures. Only two studies used a validated measure of exercise [41,46] the 6-Minute Walking Distance [50]. The majority of studies did not measure the quantity or intensity of the activity programme. It therefore proved difficult to compare the equivalent benefits of each study. We could not estimate the amount of exercise required to achieve an improvement in levels of exercise activity or mental health symptoms.

Exercise programmes resulted in only moderate or little change in measured outcomes. It is unclear why these programmes did not lead to a more significant improvement. The physical activity component of the interventions may not have been sufficiently intense. The duration of the programme may have been too short to bring about change. Delivery of the uptake of the sessions may have been inadequate to achieve a change in activity levels. The difficulties bringing about change in this population may reflect the inherent problems in this population. There are greater levels of smoking, weight problems, and co-morbid illness [51]. Individuals with greater risk factors and co-morbid physical illness may have more difficulty achieving higher levels of physical activity.

The review found that attrition levels were low with one study having no drop-out of participants. Analysis of dropout rates can give valuable information about patient characteristics and trial characteristics that affect the overall uptake of an intervention [52]. Loss to follow up after recruitment and attrition in randomised controlled trials affects the generalisability and the reliability of their results [52,53]. Studies of health-behaviour change [54] in the general population have found varying levels of attrition. Smoking cessation interventions have been found to have attrition rates of up to 49%. Attrition rates of between 27% [55] and 32.8% [56] have been observed in weight loss studies. In people with serious mental illness similar levels of attrition have been found. Khan et al. [57] found in 45 trials of antidepressants with a total of 19,000 subjects the mean drop out rate was 37%. Explanation for the low level of attrition in this review is unclear. Low drop-out rates may suggest the participation of motivated individuals willing to change their behaviour. Several studies included individuals based in hospital wards. The structured setting of a ward environment may have reduced the levels of attrition.

Despite the health problems of this population we found only 8 randomised controlled trials assessing the effectiveness of exercise programmes for people with serious mental illness. However the number of studies in this field has steadily grown over the past few years. For example, Ellis et al. [58] in a systematic review of exercise programmes in psychosis identified one randomised controlled trial [41], while Gorczynski et al. [59] found three studies [41,46,60] following a systematic search completed in December 2008. However one of these studies included the effects of yoga therapy in people with schizophrenia [60]. A recent literature review by Faulkner et al. [61] found a total of 7 randomised controlled trials, however 4 of these included yoga therapy. Faulkner et al. [61] identified one additional small study [48] which was included in the review in this paper.

It is unclear why so few studies have been conducted in this field? In our review we found that many studies were excluded as they contained additional components such as dietary advice or measures to reduce weight. The focus on physical activity alone may be being lost in interventions designed to address the current increasing general concerns about obesity and metabolic problems in this population [61]. This is in addition to a general lack of well designed studies aiming to address the health problems and risk factors in this population. For example, smoking levels in people with serious mental illness remain about two to three times levels found in the general population. However relatively few randomised trials have been conducted with the primary aim of achieving smoking cessation in this population [62].

There are strengths and limitations to the results we have presented. This review found modest changes in levels of exercise activity, but no effect on symptoms of mental health. A number of limitations need to be acknowledged. Research in this field has been so far been limited with only a small number of randomised controlled trials. These tended to be small in size and of short duration. The heterogeneity of programmes affected the impact and generalisability of studies found in the review. Studies failed to quantify the amount and intensity of exercise in their programmes. Interventions tended to use non-standardised exercise programmes and a variety of outcome measures. It proved difficult to recommend from this review the most suitable and effective programme of exercise to individuals with serious mental illness. Until further research is conducted individuals with serious mental illness should be encouraged to meet the general recommendations currently advised to the general population.

Research in the future needs to focus on methods to improve levels of exercise in individuals with serious mental illness. Programmes have proved successful in the general population. There is a need to conduct well designed randomised trials of physical activity programmes. Research needs to incorporate a standardised exercise programme and outcome assessment. The duration and intensity of interventions needs to be sufficient to achieve change in levels of physical activity, mental health symptoms, or weight. New programmes need to take into account the specific needs and potential barriers to exercise of those with serious mental illness. Levels of motivation, mental health symptoms, and weight enhancing medication add additional complexity and difficulties to this process. Potential barriers to and benefits of physical activity for people with serious mental illness have been shown in previous research. McDevitt et al. [63] found that in individuals with serious mental illness symptoms of mental illness (e.g. lack of energy or volition), medication, weight gain from medication, and safety concerns restricted their ability to be active. Johnstone et al. [64] found that limited experience of previous physical activity reduced self esteem and confidence, and lack of structure or planning to their day, limited engagement in exercise activity. In the review we described three studies identified specific barriers affecting participation in their programmes. Marzolini et al. [50] found that medical and health reasons, supervised trips and family visits, and medical appointments affected participation. Skrinar et al. [45] identified several barriers such as problems with transport, financial issues, treatment factors, and conflicting schedules with other treatment programmes affected participation. Beebe et al. [43] found similar problems with the most common reasons given for non-attendance being transportation problems (22.2%), physical illness (20.6%), and conflict with another appointment (12.7%).

For clinicians there remains no clear standardised method to improve levels of physical activity in this population. The health problems of this population continue to be highlighted in many leading publications [1]. An effective method needs to be developed in this population to reduce the persistently highs levels of cardiovascular risk and mortality.

## Conclusion

In conclusion we found that exercise programmes can lead to an improvement in exercise activity but had no significant effect on symptoms of mental health or body weight. However, it is clear that further research is needed with studies of larger size using comparable interventions and outcome measures.

## Competing interests

RP, DJS, and AP declared no competing interests. JG has received research funding from MRC, ESRC, NIHR, Stanley Medical Research Institute and has received donations of drugs supplies for trials from Sanofi-Aventis and GSK. He has acted as an expert witness for Dr Reddys.

## Authors’ contributions

RP, AP, and JG developed the research. RP conducted the research. RP and DJS conducted the analysis. RP drafted the manuscript. AP, DJS, and JG provided input and approved the final version. All authors read and approved the final manuscript.

## Pre-publication history

The pre-publication history for this paper can be accessed here:

http://www.biomedcentral.com/1471-244X/14/117/prepub
